# InGaAs FinFETs Directly Integrated on Silicon by Selective Growth in Oxide Cavities

**DOI:** 10.3390/ma12010087

**Published:** 2018-12-27

**Authors:** Clarissa Convertino, Cezar Zota, Heinz Schmid, Daniele Caimi, Marilyne Sousa, Kirsten Moselund, Lukas Czornomaz

**Affiliations:** IBM Research GmbH Zürich, Säumerstrasse 4, CH-8803 Rüschlikon, Switzerland; zot@zurich.ibm.com (C.Z.); cai@zurich.ibm.com (D.C.); sou@zurich.ibm.com (M.S.); kmo@zurich.ibm.com (K.M.); luk@zurich.ibm.com (L.C.)

**Keywords:** III-V, TASE, MOSFETs, Integration

## Abstract

III-V semiconductors are being considered as promising candidates to replace silicon channel for low-power logic and RF applications in advanced technology nodes. InGaAs is particularly suitable as the channel material in n-type metal-oxide-semiconductor field-effect transistors (MOSFETs), due to its high electron mobility. In the present work, we report on InGaAs FinFETs monolithically integrated on silicon substrates. The InGaAs channels are created by metal–organic chemical vapor deposition (MOCVD) epitaxial growth within oxide cavities, a technique referred to as template-assisted selective epitaxy (TASE), which allows for the local integration of different III-V semiconductors on silicon. FinFETs with a gate length down to 20nm are fabricated based on a CMOS-compatible replacement-metal-gate process flow. This includes self-aligned source-drain n^+^ InGaAs regrown contacts as well as 4 nm source-drain spacers for gate-contacts isolation. The InGaAs material was examined by scanning transmission electron microscopy (STEM) and the epitaxial structures showed good crystal quality. Furthermore, we demonstrate a controlled InGaAs digital etching process to create doped extensions underneath the source-drain spacer regions. We report a device with gate length of 90 nm and fin width of 40 nm showing on-current of 100 µA/µm and subthreshold slope of about 85 mV/dec.

## 1. Introduction

Compound semiconductors based on arsenides (In_1−x_Ga_x_As) [1] are considered promising candidates to replace silicon in nFETs for advanced and ultra-scaled CMOS technology nodes. These materials offer a significant advantage in terms of electron mobility compared to silicon and are suitable for low-power applications [2,3,4,5]. Nevertheless, to enable large-scale and cost-effective integration, the challenge of transferring high quality III-V material on silicon must be overcome. Recently, different strategies for III-V on silicon integration have been proposed. Strain-relaxed buffer layer growth and direct wafer bonding (DWB) [6,7], can enable large-area III-V-on-insulator substrates, as well as 3D heterogenous integration on processed substrates [8]. Selective epitaxial techniques make possible, instead, local integration of III-V crystals in pre-defined regions [9,10]. This approach can potentially reduce the costs associated with III-V substrates and simplify the integration process. Defect density can be engineered by tuning multiple aspects such as cavity geometry, nucleation seed or growth direction. Aspect-ratio trapping (ART) technique [11,12], for instance, aims to filter crystals defects propagating along (111) planes but lacking in defects confinement along the trench direction. We have previously developed an integration approach called template-assisted selective epitaxy (TASE) [13,14,15], based on the growth of different III-V materials within arbitrarily shaped oxide cavities. This technique has been employed to demonstrate various devices such as tunnel FETs [16,17] ballistic nanowires [18] as well as optically active devices [19].

In this work we demonstrate InGaAs n-FinFETs integrated on a silicon (100) substrate. Due to the presence of a buried oxide layer (BOX), our InGaAs-on-insulator devices share the benefits as SOI technologies. Furthermore, we implement here a replacement-metal-gate process (RMG) on selectively grown structures with a CMOS compatible III-V process. The fabrication flow includes n-doped InGaAs contacts regrowth as well as SiN_x_ spacers. Source-drain doped extensions are obtained by digital etching of the InGaAs channel and regrowth, in order to mitigate the access resistance increase introduced by the presence of spacers. InGaAs material quality is investigated through STEM analysis (JEOL ARM-200F, Tokyo, Japan). Electrical characteristic of FinFETs devices at two different gate length is shown, as well as the effect of scaling on the transistor subthreshold operation.

## 2. Materials and Methods

**Figure 1:**
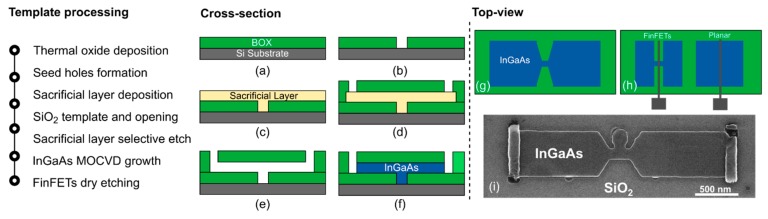
Overview of the fabrication process. (left) process flow up to the InGaAs fin formation. Cross-section schematic of (**a**) thermal oxide formation on silicon substrate, (**b**) patterning and opening of seed area in the oxide layer, (**c**) sacrificial layer deposition, (**d**) oxide template deposition and patterning of opening areas, (**e**) selective removal of sacrificial material, (**f**) III-V MOCVD growth. Top-view schematic of (**g**) as-grown InGaAs structure after SiO_2_ template strip and (**h**) planar and FinFETs structures after dry etching. (**i**) SEM top-view image of grown InGaAs structure from a seed positioned off-center.

**Figure 2:**
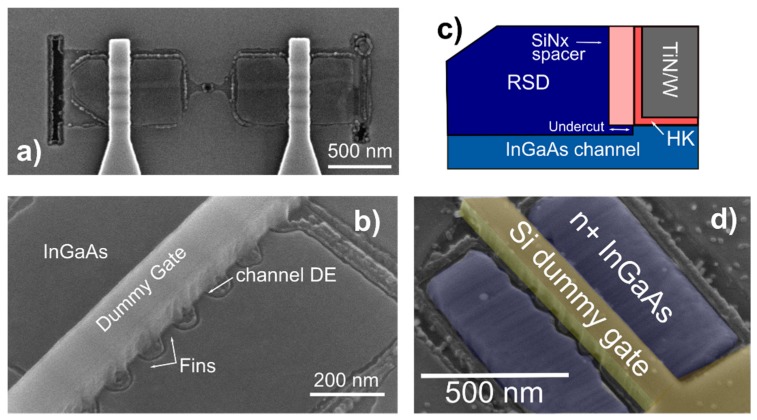
Device fabrication steps. (**a**) Top-view SEM image of InGaAs FinFET device after dummy gate patterning and dry etch. Fins are visible underneath the gate. (**b**) SEM picture showing InGaAs fins after digital etching (DE). The channel recess underneath the dummy gate is performed to allow for doped extensions regrowth. This is schematized in (**c**) with a cross-section drawing zooming on the channel/RSD interface. (**d**) InGaAs transistor SEM top-view after MOCVD RSD growth.

The InGaAs devices are fabricated by TASE. First, a SiO_2_ thermal oxide layer 50 nm thick is deposited on a silicon wafer (Figure 1a). Seed-area openings, with a diameter of 50 nm, are patterned on the oxide layer by e-beam lithography using PMMA resist (Figure 1b). A sacrificial layer 30 nm thick is then deposited on top of the BOX. Thickness and morphology of this layer will define the features of the final grown semiconductor structure. Next, the sacrificial material is patterned by e-beam lithography and the structures to be transferred into III-V are dry etched (Figure 1c). A second oxide layer, 100 nm thick, referred to as the oxide template, is deposited above the patterned structures. Afterward, openings are patterned by e-beam lithography using PMMA resist and vias are dry etched down to the sacrificial material layer (Figure 1d). The latter is therefore selectively etched, exposing the silicon seed that was previously formed (Figure 1e). Thus, the as-formed cavities contain a small seed opening to the silicon substrate. The nucleation point is perpendicular to the growth direction (Figure 1f), enabling efficient defect filtering in multiple directions [15]. A short dip in diluted HF is performed to remove the native oxide formed on the Si seed. The sample is then immediately loaded into a metal–organic-chemical-vapor-deposition (MOCVD) reactor and In_0.53_Ga_0.47_As is grown into the patterned structures. Trimethylgallium (TMGa), trimethylindium (TMIn) and tertiarybutylarsine (TBAs) are used as precursors for the InGaAs growth, performed at 550 °C. The growth is geometrically confined into the formed template cavities. Afterwards, the template is removed by a combination of dry reactive ion etching (RIE) and HF wet etching. The replacement-metal-gate (RMG) process starts with the deposition and patterning of a dummy gate (Figure 2a). 3 nm Al_2_O_3_ (working as etch-stop layer) and 150 nm amorphous silicon are deposited and patterned by using HSQ resist. The smallest physical gate length measured is 20 nm. The amorphous silicon is dry etched by inductively-coupled-plasma (ICP) RIE with an optimized process for vertical sidewalls. The silicon etching stops on the Al_2_O_3_ layer. Next, 4 nm thick SiN_x_ spacers are deposited and dry etched by RIE. The Al_2_O_3_ on the InGaAs areas not covered by the dummy gate is then removed by wet etching in HF solution. As schematized in Figure 2c, the InGaAs channel is recessed underneath the sidewall spacers. This is achieved by several cycles of III-V digital etching (DE). The cycle consists in placing the sample in ozone atmosphere for 8 min and subsequently removing the as-formed III-V oxide in an HCl dilution with de-ionized water (1:10) for 15 s. The estimated etch rate per DE cycle is about 1.5 nm. Following, the sample is immediately loaded in the MOCVD reactor to perform raised-source-drain (RSD) epitaxy of n-doped In_0.53_Ga_0.47_As. The dopant used is Sn and the estimated doping level is 1 × 10^19^ cm^−3^. A SEM (Hitachi SU8000, Tokyo, Japan) micrograph of the InGaAs device after RSD epitaxy is shown in Figure 2d. RSD epitaxy is followed by an inter-layer dielectric deposition (250 nm of SiO_2_) that is subsequently planarized by chemical mechanical polishing (CMP). The oxide planarization allows to expose the top part of the dummy gate that is then removed by a selective XeF_2_-based etch. The dummy high-k oxide is hence removed by HF etching and the sample is immediately loaded in the ALD chamber for the gate-stack deposition. The gate-stack consists of 5 nm Al_2_O_3_/HfO_2_ bilayer high-k dielectric and TiN metal-gate. Right after, 150 nm of W are sputtered and planarized by CMP. A second ILD (Interlayer dielectric) layer (50 nm of SiO_2_) is deposited and vias down to source/drain/metal are opened and filled with tungsten. Metal pads are patterned with negative e-beam resist and dry etched with RIE. Prior to measuring the devices, a forming gas anneal (FGA) at 300 °C is performed.

## 3. Results and Discussion

**Figure 3:**
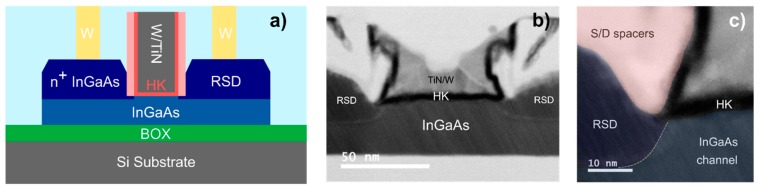
(**a**) STEM structural analysis. Cross-section schematic of completed InGaAs FET device, after final M1 metallization step. (**b**) STEM cross-section image for a device with LG = 60 nm. The RSD/channel interface is clearly distinguishable due to difference in indium content between the two. A false-colored zoomed view on the sidewall spacer/HK/RSD/channel interface is shown in (**c**).

**Figure 4:**
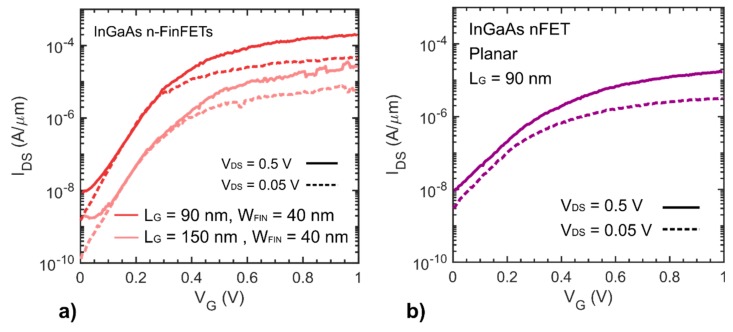
(**a**) Transfer characteristic of InGaAs FinFET device with L_G_ = 90 nm and L_G_ = 150 nm. Fin width is in both cases 40 nm. The shorter gate length device shows an on-current of 100 µA/µm and SS of 85 mV/decade. The gate leakage current (not shown) is at the limit of the measurement equipment, less than 1 pA. (**b**) Representative transfer characteristic of a planar InGaAs device with L_G_ = 90 nm.

**Figure 5:**
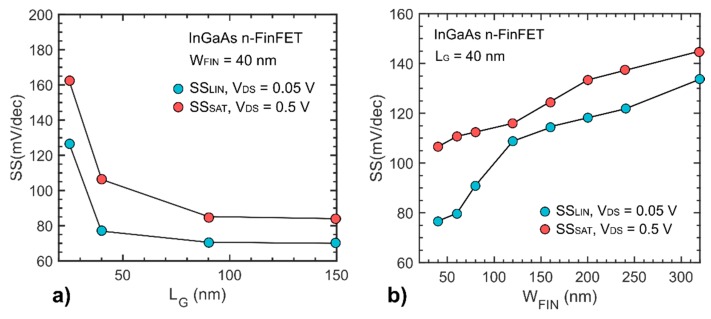
(**a**) Subthreshold slope values versus gate length for devices with W_FIN_ = 40 nm. For LG smaller than 40 nm, short channel effects start dominating the device behavior. (**b**) Average SS versus W_FIN_ for devices with L_G_ = 40 nm. The trend indicates that subthreshold performance benefits from further reducing fin width.

A schematic of the device cross-section is shown in Figure 3a with the corresponding STEM cross-sections of the finished device shown in Figure 3b and a magnified view of the spacer/RSD/channel corner (Figure 3c). The RSD and channel interface are clearly distinguishable. This is possibly due to an undesired drift in indium content between the two growth runs. The presence of planar stacking faults along a (111) plane are visible as well, originating from the growth process. Extensive study of growth dynamic and defects in InGaAs structures grown by TASE can be found in [20]. Electrical I_DS_-V_G_ characteristics of 90 nm and 150 nm gate length devices as measured after FGA are shown in Figure 4. The shorter gate length (L_G_) device with fin width of W_FIN_ = 40 nm, exhibits 85 mV/dec and 95 mV/dec subthreshold slopes (SS) at V_DS_ = 0.05 V and V_DS_ = 0.5 V, respectively. SS is overall improved compared to devices reported in [15], where a SS of 190 mV/dec is achieved for L_G_ = 100 nm and W_FIN_ = 50 nm. The same device shows a transconductance peak value in saturation of 350 µS/µm and a maximum transconductance efficiency around 20 V^−1^ at 0.3 V. The R_ON_ of this device, measured from the output characteristics, is about 1 kΩ·µm. Compared to our previously reported InGaAs FinFETs [15] fabricated using a similar scheme, we achieved significant improvement in off-state performance as well as better I_ON_/I_OFF_ = 10^4^. This can be attributed to the presence of extended source-drain RSD contacts reaching underneath the spacer region, limiting the access resistance increase introduced by ungated channel area. The short L_G_ device shows an I_ON_ = 100 µA/µm, at fixed I_OFF_ = 100 nA/µm at V_DS_ = 0.5 V (Figure 4a) and a V_GS_ voltage swing of 0.5 V, which is the intended bias point for III-V MOSFETs. The two reported L_G_, with a comparable slope, illustrate the positive impact of scaling on the on-state performance. The transfer characteristic of a planar device is shown in Figure 4b. For the same L_G_, planar devices show higher SS due to degraded electrostatic control, and lower maximum on-current due to smaller normalized gate width compared to FinFET devices. Figure 5a, shows average SS values versus L_G_ for FinFETs with W_FIN_ = 40 nm. For L_G_ smaller than 40 nm, both linear and saturation SS increase substantially, due to short-channel effects. In Figure 5b, SS versus W_FIN_ for fixed L_G_ is plotted, showing that SS will benefit from further fin width scaling, due to improvement of the electrostatic control. The performance improvement achieved in this work compared to previously reported devices [15] are attributed to the use of an RMG scheme instead of a gate-first (GF) one. Here, the high-k/channel interface is less exposed to thermally induced degradation from the high-temperature RSD growth step, resulting in a lower density of interface traps.

## 4. Conclusions

In this work, we have demonstrated InGaAs n-FinFETs devices using a novel III-V integration technique based on TASE. A full RMG process flow was developed including an improved RDS process with underlapping extensions. Devices with gate length of 90 nm and fin width of 40 nm show on-current of 100 µA/µm and subthreshold slope of about 85 mV/dec, demonstrating good electrostatic control. The strong off-state performance is due to the introduction of source-drain sidewall spacers combined with doped extensions achieved by digital etching. Results indicate that future performance improvements could be achieved by further scaling of gate length and fin width.
